# Enhancement of Long-Range Surface Plasmon Excitation, Dynamic Range and Figure of Merit Using a Dielectric Resonant Cavity

**DOI:** 10.3390/s18092757

**Published:** 2018-08-22

**Authors:** Phitsini Suvarnaphaet, Suejit Pechprasarn

**Affiliations:** College of Biomedical Engineering, Rangsit University, Pathum Thani 12000, Thailand; phitsini.s@rsu.ac.th

**Keywords:** long-range surface plasmon polariton, short-range surface plasmon polariton, dielectric resonant cavity, multiple reflections, plasmonic, surface plasmon resonance sensor, instrumentation

## Abstract

In this paper, we report a theoretical framework on the effect of multiple resonances inside the dielectric cavity of insulator-insulator-metal-insulator (IIMI)-based surface plasmon sensors. It has been very well established that the structure can support both long-range surface plasmon polaritons (LRSPP) and short-range surface plasmon polaritons (SRSPP). We found that the dielectric resonant cavity under certain conditions can be employed as a resonator to enhance the LRSPP properties. These conditions are: (1) the refractive index of the resonant cavity was greater than the refractive index of the sample layer and (2) when light propagated in the resonant cavity and was evanescent in the sample layer. We showed through the analytical calculation using Fresnel equations and rigorous coupled wave theory that the proposed structure with the mentioned conditions can extend the dynamic range of LRSPP excitation and enhance at least five times more plasmon intensity on the surface of the metal compared to the surface plasmon excited by the conventional Kretschmann configuration. It can enhance the dip sensitivity and the dynamic range in refractive index sensing without losing the sharpness of the LRSPP dip. We also showed that the interferometric modes in the cavity can be insensitive to the surface plasmon modes. This allowed a self-referenced surface plasmon resonance structure, in which the interferometric mode measured changes in the sensor structure and the enhanced LRSPP measured changes in the sample channel.

## 1. Introduction

Surface Plasmon Resonance (SPR) has been a powerful scientific tool for biological protein kinetic studies [1,2,3,4]. The SPR sensing platforms are usually implemented based on the optical configuration proposed by Kretschmann-Raether in 1968 [5,6]. That same year, Otto also proposed an optical configuration that allowed the SPR to be excited on a bulk gold surface [7]. The Otto configuration has not been exploited much in biosensing applications since the structure requires a very narrow spacing gap (less than a wavelength spacing height) between the coupling prism and the bulk plasmonic metal.

It has been very well established that double plasmonic metal interfaces such as: (1) insulator-metal-insulator (IMI) structures [8] or (2) metal-insulator-metal (MIM) structures [9,10,11] can support two plasmonic modes, so-called, long-range surface plasmon polaritons (LRSPP) and short-range surface plasmon polaritons (SRSPP) [12]. These LRSPP and SRSPP modes have been experimentally verified by several groups including Lee et al. [13] and Slavík et al. [14]. The LRSPP mode has several preferable features over the SRSPP mode including (1) it can be excited at a lower incident *k*-vector (lower angle and lower incident wavelength) and (2) it has a higher figure of merit (*FoM*). The *FoM* is a quantity used to measure the detection performance, relative to a ratio of the sensitivity to the full-width half maximum (FWHM) of the reflectance dip position in SPR biosensing platform. This is due to the change of the SPR signal upon local refractive index changes. Thus the narrow reflection dip and enhancement of the sensitivity improves the *FoM* of SPR sensors [15]. The enhanced *FoM* has proven itself very useful in biosensing platforms and applications. Khan et al. [16] reported the implementation of an interdigitated capacitor (IDC)-based glucose biosensor with high detection sensitivity of 10^−7^ refractive index units (RIU) and wide dynamic range for fast response and recovery in which the limit of detection reached nanomolar concentration. Zhen et al. [17] has reported an ultra-sensitive plasmonic biosensor having the detection sensitivity down to 10^−18^ molar concentration of ssDNA sensing by nanostructures hybridized gold thin film. Yu et al. [11] have recently proposed the side-coupled Fabry-Perot cavity as a resonator for ultrahigh wavelength selection. Wu et al. [18] have reported the theoretical performance of MIM plasmonic structures with a ring resonator.

The LRSPP and SRSPP modes occur due to a hybridization between the two bound SPR modes on two metal surfaces. This lower excitation angle, of course, reduces the requirement of using high numerical aperture (NA) objective lens to excite the LRSPP [19]. The LRSPP does, however, still have two main issues. Firstly, it does have a very limited dynamic range since it can only be excited when refractive indices sandwiching the plasmonic metal layer are similar. Secondly, although the LRSPP reflectance dip is much narrower than the conventional Kretschmann SPR, the dip sensitivity in refractive index sensing is much less. The longer propagation length of LRSPP can offer a larger detection area of the binding sample region allowing the LRSPP to be more sensitive than the conventional SRSPP [12].

**Figure 1:**
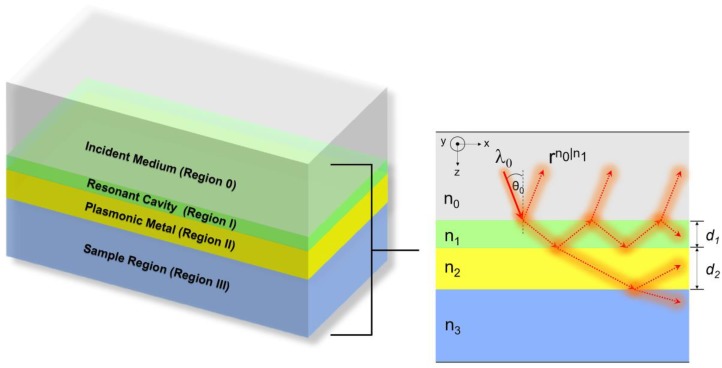
The proposed IIMI structure. The incident medium (Region 0) was a semi-infinite glass substrate with refractive index *n*_0_ serving as an incident medium. The resonant cavity (Region I) was made of a dielectric layer with refractive index *n*_1_ and thickness *d*_1_. The plasmonic medium (Region II) was a noble metal layer with refractive index *n*_2_ and thickness *d*_2_. The sample medium (Region III) was also the dielectric layer with refractive index *n*_3_ which was the sample channel of the proposed SPR sensor.

The insulator-insulator-metal-insulator (IIMI) structure for LRSPP excitation was first introduced by Abelès and Lopez–Rios in 1974 [20]. The major differences for the IIMI structure [13,14,20] reported by others and this paper are that we are interested in the IIMI as shown in Figure 1 under certain conditions which were: (1) the refractive index of the dielectric resonant cavity (*n*_0_) was larger than the refractive index of the sensing sample region (*n*_3_); (2) the *k*-vectors where the light propagated in the dielectric cavity were evanescent in the sample region. 

The thickness of the dielectric cavity considered here ranged from 0.5 to 4 μm, which is much thicker than the LRSPP structures reported in the literature. Thus the proposed structure served as a resonator allowing multiple reflections inside the cavity and interferometric modes. By matching the *k*-vectors of the resonant cavity and the LRSPP, these conditions can enhance the properties of the LRSPP excitation, dynamic range and *FoM* in SPR sensing applications. To the best of the authors’ knowledge, these features of the LRSPP excited through the dielectric resonant cavity have never been reported before.

## 2. Structure and Simulation Methods

### 2.1. Simulation Methods

The multi-layer type structure shown in Figure 1 was analysed using a transfer matrix approach for Fresnel equations [21]. The following variables were used throughout the paper: *r_p_* and *t_p_* are the reflection coefficient and transmission coefficient for p-polarization and s-polarization, respectively. Rigorous coupled wave theory [22] has been implemented to determine the mode profiles in the structure and stored optical power per unit length in the resonant cavity. *E_x_*, *E_y_* and *E_z_* are the electric fields along the *x*-, *y*- and *z*-axes, respectively. *H_x_*, *H_y_* and *H_z_* are the magnetic fields along the *x*-, *y*- and *z*-axes, respectively. The axes were defined as indicated in Figure 1.

### 2.2. Terms and Definitions

There are several definitions for figure of merit (*FoM*) [23]. The commonly used *FoM* that is defined to evaluate the dip position measurement in SPR systems [15], which is also used in this paper and defined as:

(1) $$FoM=\frac{S}{\mathrm{FWHM}}$$

where *FoM* is the figure of merit for dip position measurement.

*S* is the sensitivity term. In the SPR biosensing platforms, the sensitivity is herein defined in term of *dk_x,resonance_*/*dn_sample_* for bulk sensitivity where the *k_x,resonance_* is the *k*-vector position of the resonant mode along the *x*-axis and the *n_sample_* is the refractive index of the bulk sample medium.

FWHM is the full-width half maximum of the reflectance dip. The width of the dip is measured at 0.5 intensity level of the reflectance curve. Dynamic range (*D*) is defined as the range of refractive indices that the plasmonic reflectance dip intensity is below 0.25. The illumination *k*-vector in this paper is presented as a normalized *k*-vector, $\frac{k_{x}}{k_{free-space}}=n_{o}\sin\theta_{0}$ or numerical aperture (NA).

## 3. Results and Discussion

### 3.1. LRSPP and SRSPP

**Figure 2:**
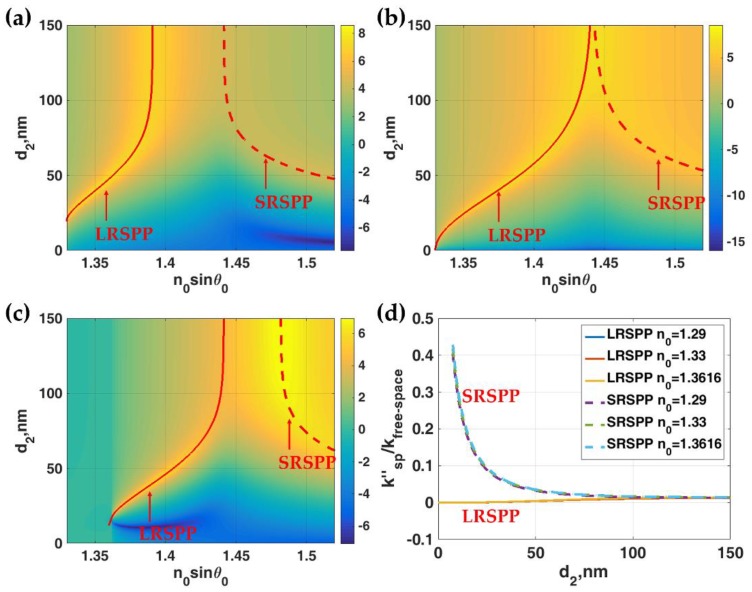
Comparison of coupling strength, *ln|r_p_|*^2^, of LRSPP and SRSPP in the case where *n*_0_ = *n*_1_ and *d*_1_ = 0 nm considered as pure plasmonic modes for (**a**) *n*_0_ = *n*_1_ = 1.29; (**b**) *n*_0_ = *n*_1_ = 1.33 and *n*_0_ = *n*_1_ = 1.3616. Other parameters are *λ* = 633 nm, *n*_2_ = 0.18344 + 3.4332*i* and *n*_3_ = 1.33; (**d**) Normalized attenuation coefficients $k_{sp}^{"}/k_{free-space}$ for the cases in (**a**–**c**). The solid red curves in (**a**–**c**) were the solutions to Equation (2) for LRSPP mode and the dashed red curves are the solutions for SRSPP mode.

Before looking at the IIMI plasmonic structure, let us consider the case where we only had pure plasmonic modes in a three homogenous-layer structure, when either *d*_1_ was treated as 0 nm or *n*_0_ equalled *n*_1_. The structure was a thin plasmonic gold layer with thickness *d*_2_ sandwiched by two semi-infinite dielectric media with refractive indices of *n*_0_ and *n*_3_. Gold has been chosen in this study since it is suitable for biological sensor fabrication because gold is chemically stable, inductive and non-toxic to biological samples [3,24]. The refractive index of gold is 0.18344 + 3.4332*i* at 633 nm free-space wavelength (*λ*) extracted from Johnson and Christy [25]. Note that we have compared and discussed the performance of silver with refractive index of 0.056206 + 4.2776*i* [25] as reported and shown in Figure S1 and Table S1 in the Supplementary Information (SI). The silver results tell the same story as the gold results. The results are therefore omitted from the main manuscript. Figure 2a–c shows *ln*(|*r_p_*|^2^) for different values *n*_0_ ranging from 1.29 (Teflon™ AF2400 [26]), 1.33 and 1.3616 (potassium fluoride: KF [27]), respectively, when *n*_3_ was fixed at 1.33. It is important to point out that the *n*_0_*sinθ*_0_ range in the calculation was 1.33 to 1.52. This made the *sinθ*_0_ greater than 1 indicating that the SPR modes shown here were excited by an evanescent wave and the structure required a higher coupling index medium or a grating to satisfy the *k*-vector condition. Of course, if another higher refractive coupling layer had been added to the structure, this would form an IIMI structure. The interferometric modes in the IIMI can unavoidably interact with the plasmonic modes explained in the later section. These Fresnel calculations have enabled us to identify the mode coupling strength and the *k*-vector positions for the pure SRSPP and pure LRSPP modes without any influence from the coupling layer. It has been very well established that the LRSPP exists under certain conditions, which are (1) the refractive indices, which sandwich the plasmonic metal, have to be similar [12] and (2) the plasmonic metal thickness is usually thinner than the metal used in Kretschmann configuration. Figure 2b showed that when the *n*_0_ and *n*_3_ refractive indices were perfectly matched, the coupling strength for the both LRSPP (symmetric mode) and SRSPP (asymmetric mode) were similar. On the other hand, when they were mismatched, the LRSPP had a stronger coupling compared to the SRSPP when *n*_0_ was lower than *n*_3_ as shown in Figure 2a in a comparison with Figure 2c. The separation in *k*-vector space between the two SPP modes was wider when the plasmonic gold was thin. On the other hand, when the gold was thicker than 75 nm the two SPP modes combined to a single mode. The LRSPP *k*-vector appeared at a larger *k*-vector value with the larger *n*_0_ refractive index. The thickness of the gold *d*_2_ and the refractive index sandwiching the plasmonic gold will be employed as a key parameter to vary the *k*-vector positions of the two plasmonic modes in the next section.

Stegeman and Burke [28] have reported a dispersion relation for the two plasmonic modes for asymmetric metal waveguide [29], which is given by:

(2) $$\tanh(k_{z2}d_{2})(\varepsilon_{0}\varepsilon_{3}k_{z2}^{2}-\varepsilon_{2}^{2}k_{z0}k_{z3})-\varepsilon_{2}k_{z2}(\varepsilon_{0}k_{z3}+\varepsilon_{3}k_{z0})=0$$

where *k_z_*_0_, *k_z_*_2_, and *k_z_*_3_ are the *k*-vectors along the *z*-axis in the region 0, II and III. $\varepsilon_{0}$, $\varepsilon_{2}$ and $\varepsilon_{3}$ are the permittivity of the region 0, II and III. These permittivity values can be determined by $\varepsilon_{0}=n_{0}^{2}$, $\varepsilon_{2}=n_{2}^{2}$ and $\varepsilon_{3}=n_{3}^{2}$.

Equation (2) allows us to solve for complex surface plasmon *k*-vector: $k_{x}=k_{sp}=k_{sp}^{\prime }+ik_{sp}^{\prime \prime }$, where *k_sp_* is the complex surface plasmon *k*-vector, where $k_{sp}^{\prime }$ is the real part of the complex surface plasmon *k*-vector and $k_{sp}^{\prime \prime }$ is the attenuation coefficient. *k_x_* is the *k*-vector along the *x*-axis, which is given by $\frac{2\pi n_{0}}{\lambda }\sin\theta_{0}$. $\theta_{0}$ is the incident angle in the incident medium as shown in Figure 1. The normalized $k_{sp}^{\prime }$ solutions to the Equation (2) were shown in Figure 2a–c as solid red curves for the LRSPP and dashed red curves for the SRSPP. The normalized $k_{sp}^{\prime }$ solutions to the Equation (2) for the cases described in Figure 2a–c is shown in Figure 2d. The attenuation loss for the LRSPP was much lower than the SRSPP. It was dependent mostly on the thickness of the metal, not the coupling refractive index.

### 3.2. IIMI Structure for LRSPP Excitation through Evanescent Wave Coupling $n_{1}\leq n_{3}$

**Figure 3:**
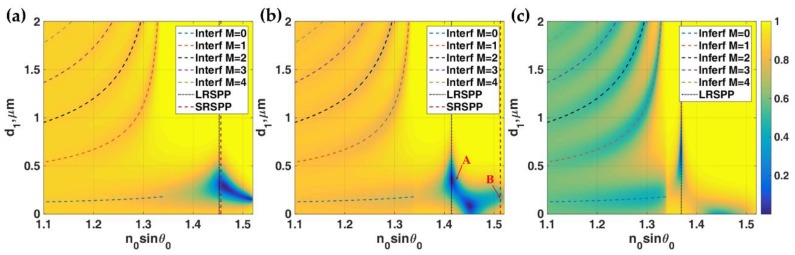
Responses showed |*r_p_*|^2^ for *λ* = 633 nm *n*_0_ = 1.52, *n*_1_ = 1.34 with thickness *d*_1_ and *n*_2_ = 0.18344 + 3.4332*i* with thickness *d*_2_, *n*_3_ = 1.34 (**a**) *d*_2_ = 150 nm (**b**) *d*_2_ = 60 nm and (**c**) *d*_2_ = 30 nm. The colored curves shown in these figures were the interferometric mode positions calculated using Equation (3) and the two plasmonic modes calculated using Equation (2). ‘A’ and ‘B’ were labeled as plasmonic modes calculated using Equation (2). ‘C’, ‘D’ and ‘E’ are labelled as interferometric modes.

The coupling refractive index of 1.3616 was not sufficient to excite the surface plasmon modes. It was, therefore, essential to provide a higher refractive index coupling media such as the glass substrate with the refractive index of 1.52. This glass substrate did not only provide the sufficient *k*-vector to excite the surface plasmons, but it also provided the interferometric modes and the second dielectric layer can also serve as a resonant cavity. These interference fringes were formed by the direct reflection from the first interface between the region 0 and the region I, $r_{p}^{{}_{{}^{n_{{}_{0}}|n_{{}_{1}}}}}$, and the multiple reflected beams as depicted in Figure 1. For the $n_{1}\leq n_{3}$ case, here *n*_1_ of 1.34 was calculated as an example. In this case, all interferometric mode *k*-vectors appeared below the *k*-vectors of the two SP modes as shown in Figure 3. 

These interferometric mode positions can be calculated using the asymmetric Fabry Perot cavity resonant mode number [30], which is given by:

(3) $$2k_{z1}+Phase(r_{p}^{{}_{{}^{n_{{}_{1}}|n_{{}_{2}}|n_{{}_{3}}}}})+Phase(r_{p}^{{}_{{}^{n_{{}_{1}}|n_{{}_{0}}}}})=2M\pi$$

where $Phase(r_{p}^{{}_{{}^{n_{{}_{1}}|n_{{}_{0}}}}})$ and $Phase(r_{p}^{{}_{{}^{n_{{}_{1}}|n_{{}_{2}}|n_{{}_{3}}}}})$ are the phase of reflection coefficients for p-polarization for the light travelling the region I to the region 0 and the region I to the region II and the region III, respectively. *M* is the resonant mode number, *M* = 0, 1, 2, 3, ….

**Figure 4:**
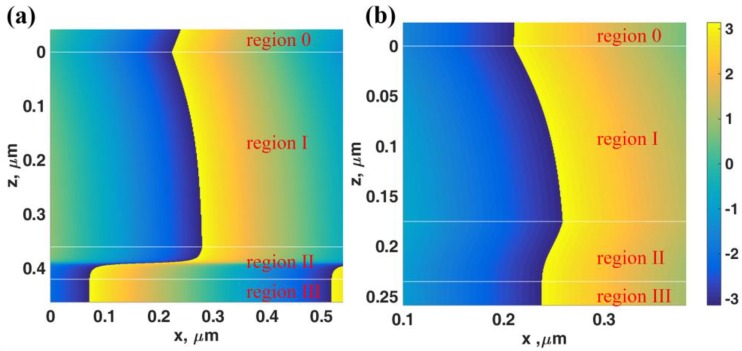
Phase profiles of *E_x_* in *rad* for (**a**) LRSPP mode labelled ‘A’ in Figure 3b: *n_0_sinθ*_0_ = 1.4156, *n*_1_ = 1.34 with *d*_1_ = 360 nm, *n*_2_ = 0.18344 + 3.4332*i* with *d*_2_ = 60 nm and *n*_3_ = 1.34 and (**b**) SRSPP mode labelled ‘B’ in Figure 3b: *n_0_sinθ*_0_= 1.513, *n*_1_ = 1.34 with *d*_1_ = 175 nm, *n*_2_ = 0.18344 + 3.4332*i* with *d*_2_ = 60 nm and *n*_3_ = 1.34.

The 0th order did not have a cut-off *k*-vector unlike the other higher order modes [31] and it can excite the LRSPP mode. The interferometric mode positions matched very well with the Fresnel simulations. The interferometric dips were not very deep indicating that the gold layer cannot provide sufficient loss for the interferometric modes. Figure 3a–c shows |*r_p_*|^2^ responses of the IIMI structure with different gold thicknesses. The two plasmonic modes were excited through attenuated total internal reflection (ATR) evanescent wave, where the light was evanescent in the region I after *n_0_sinθ*_0_ of 1.34. The two plasmonic mode *k*-vectors changed when the gold thickness changed as predicted from Figure 3. The dielectric cavity height *d*_1_ played a crucial role in the plasmonic mode coupling. It cannot be too high because of the short penetration of the evanescent wave. Figure 4 showed the phase transition through the gold layer of the two plasmonic modes labelled ‘A’ and ‘B’ in Figure 3b. The LRSPP had an anti-phase pattern at the two interfaces of the gold layer as shown in Figure 4a whereas the SRSPP had an in-phase phase pattern at the gold interfaces.

### 3.3. LRSPP Excited by the Dielectric Resonator $n_{1}>n_{3}$

**Figure 5:**
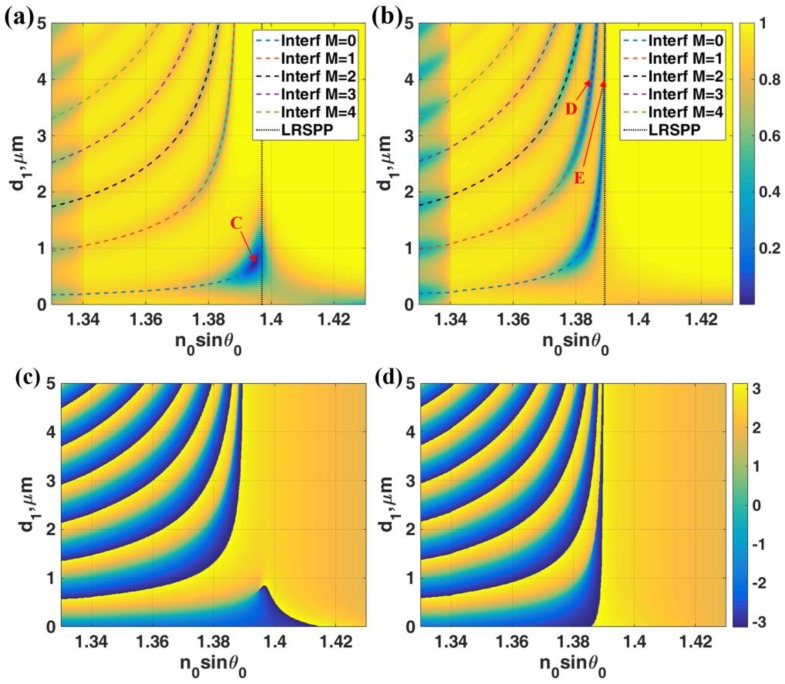
Simulation results for λ = 633 nm, *n*_0_ = 1.52, *n*_1_ = 1.39 with thickness *d*_1_ and *n*_2_ = 0.18344 + 3.4332*i* with thickness *d*_2_ and *n*_3_ = 1.34. (**a**) |*r_p_*|^2^ when *d*_2_ = 30 nm and (**b**) |*r_p_*|^2^ when *d*_2_ = 20 nm (**c**) *Phase(r_p_)* in *rad* when *d*_2_ = 30 nm and (**d**) *Phase(r_p_)* in *rad* when *d*_2_ = 20 nm. The colored curves shown in (**a**,**b**) are the interferometric mode positions calculated using Equation (3) and the LRSPP mode

In this section, let us look at the case where $n_{1}>n_{3}$. There was a range of *k*-vectors that the light was a propagating wave in the region I and evanescent in the region III. The range was the *k*-vectors with *n_0_sinθ_0_* between *n*_3_ and *n*_1_. Figure 5a,b showed |*r_p_*|^2^ for the IIMI structure with *n*_0_ = 1.52, *n*_1_ = 1.39 (lithium fluoride: LiF [27]) with thickness *d*_1_ and *n*_2_ = 0.18344 + 3.4332*i* with thickness *d*_2_ and the last dielectric medium *n*_3_ = 1.34 for different gold layer thicknesses *d*_2_ = 30 nm and *d*_2_ = 20 nm, respectively. These figures were the cases when before the LRSPP *k*-vector matched to the range of interferometric mode *k*-vectors and when they matched.

Consequently, the interferometric modes dissipated the light power more efficiently through the plasmon coupling and appeared as very deep and narrow reflectance dips at 0 intensity level as shown in Figure 5b. It is interesting to note that the LRSPP excited by the 0th order interferometric mode is, of course, excited by a propagating wave mode inside the region I. Therefore the LRSPP can be excited at any cavity thickness *d*_1_ as if the thickness allows the interferometric mode. This makes the device conveniently practical to fabricate. Not only the 0th order (*M* = 0) interferometric mode can excite the LRSPP, but also the other higher order interferometric modes if they were within the LRSPP *k*-vector range as shown in Figure 5b. In addition to the 633 nm wavelength, the other wavelengths of SPR excitation in the near-infrared region, i.e., 785 nm and 1024 nm were also presented and discussed in the SI.

**Figure 6:**
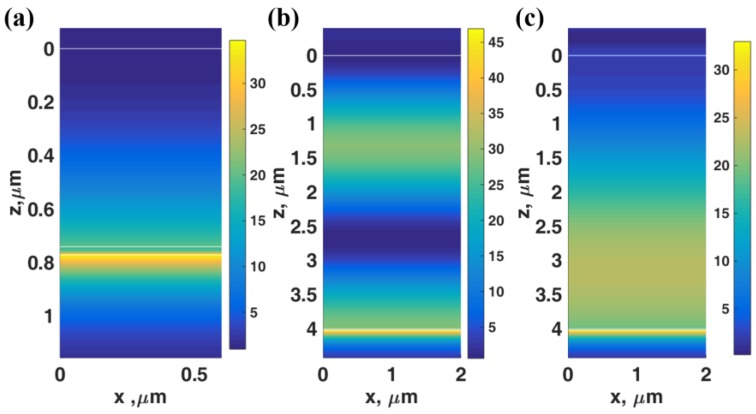
Field distributions of *|H_y_|*^2^ for (**a**) the LRSPP excited by the evanescent wave coupling labelled ‘C’ in Figure 5, (**b**) the LRSPP excited by the 1st order (*M* = 1) labelled ‘D’ in Figure 5b and (**c**) the LRSPP excited by the 0th order (*M* = 0) labelled ‘E’ in Figure 5b.

Figure 6a shows the *|H_y_|*^2^ field distributions of the LRSPP excited by the evanescent wave coupling labelled ‘C’ in Figure 5a. There was no standing wave inside the resonant cavity only the re-radiated leaky LRSPP wave leaked their power back through the cavity. Figure 6b,c shows the *|H_y_|*^2^ field distributions of the LRSPP excited by the 1st order (*M* = 1) and the 0th order (*M* = 0) interferometric modes labelled ‘D’ and ‘E’ in Figure 5b. There was the 1st order TM_1_ guided mode pattern inside the resonant cavity in Figure 6b and the fundamental 0th order mode TM_0_ guided mode pattern in Figure 6c. The sharpness LRSPP dips excited by the resonant cavity depended on the cavity thickness *d*_1_. Figure 5c,d shows the phase profiles of Figure 5a,b. The gradient of phase transitions for the interferometric modes depended strongly on the thickness of the resonant cavity. For the cavity thickness less than 1 μm, the phase gradients of all the interferometric modes were shallower than the LRSPP as shown in Figure 5a and became much sharper when the thickness increased. This feature allowed us to excite the LRSPP with the different sharpness of the reflectance dip, in other words, it allowed us to vary the FWHM of the LRSPP mode. We will illustrate that not only the FWHM can be adjusted by the cavity thickness, but it also gives different dip refractometric sensitivity. These will be fully quantified in the next section.

For fabricating consideration, the structure with such thin gold film around 20 nm to 30 nm, the gold films are likely to have some defects such as gold islands [32]. There are several research groups working on ultra-thin gold film fabrication. Kossoy [33] have achieved an artifact-free 5.4 nm gold film. Another issue that we need to consider is that for all the simulations presented in this paper, the gold layers were treated as a uniform bulk gold layer. There is no standard threshold and criteria for the thinnest thickness gold film that can be still treated as bulk gold. There are several research articles reporting an abnormal optical transmission through a thin gold film when the thickness is below 15 nm [34,35]. Laref et al. [36] have reported that the complex permittivity of gold does depends on the thickness of the gold film when the gold film is ultrathin <7 nm. The gold layer needs to be treated as anisotropic material. The complex permittivity can no longer be approximated by Drude’s model. This ultrathin layer is not in the range of our study. Olmon [37] have reported with experimental validation that the gold film thicker than 20 nm can be treated as a bulk-like material which is suitable for the range of gold thicknesses used in this proposed study.

### 3.4. Key Properties of LRSPP Excited in IIMI Structure

In this section, the sensitivity (S), full-width half maximum (FWHM), dynamic range (D) and figure of merit (*FoM*) for different LRSPP modes were quantified against the conventional SPR in Kretschmann configuration. Here the refractive indices were varied from 1.34 to 1.45 in order to quantify the sensitivity of the SPR sensor. This refractive index range covered a typical range for biological samples refractive indices [38,39]. Having explained above that the LRSPP can be excited through the evanescent wave coupling and the resonator interferometric modes.

For the Kretschmann SPR configuration, the plasmonic dips appeared at higher *k*-vector compared to the other cases presented in this section. The *S* for the Kretschmann SPR was 1.07573 for *d*_1_ of 48 nm. The maximum NA of 1.52 can only accommodate the refractive index of 1.39833 before the dip falls off the NA. One way to get around this, of course, to use a higher refractive index coupling such as NA of 1.65 [40] with coupling oil with a refractive index of 1.78 and with specially made of a sapphire coverslip [41]. This is not convenient for general biological measurements since the cover glass is very expensive and the immersion is also quite toxic and volatile. The *FoM* for the Kretschmann SPR is 22.93443.

**Figure 7:**
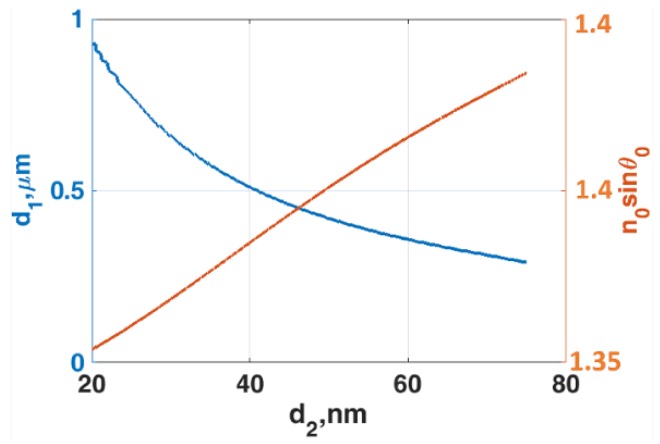
The optimal operating parameters for LRSPP excitation by evanescent wave coupling when *n*_0_ = 1.52, *n*_1_ = 1.34, *n*_2_ = 0.18344 + 3.4332*i* and *n*_3_ = 1.34 for different thicknesses of gold *d*_2_ ranging from 20 to 75 nm. The blue curve shows the cavity thickness *d*_1_ where the lowest intensity dip occurs. The red curve showed the *n*_0_*sinθ*_0_ positions where the LRSPP excited.

**Figure 8:**
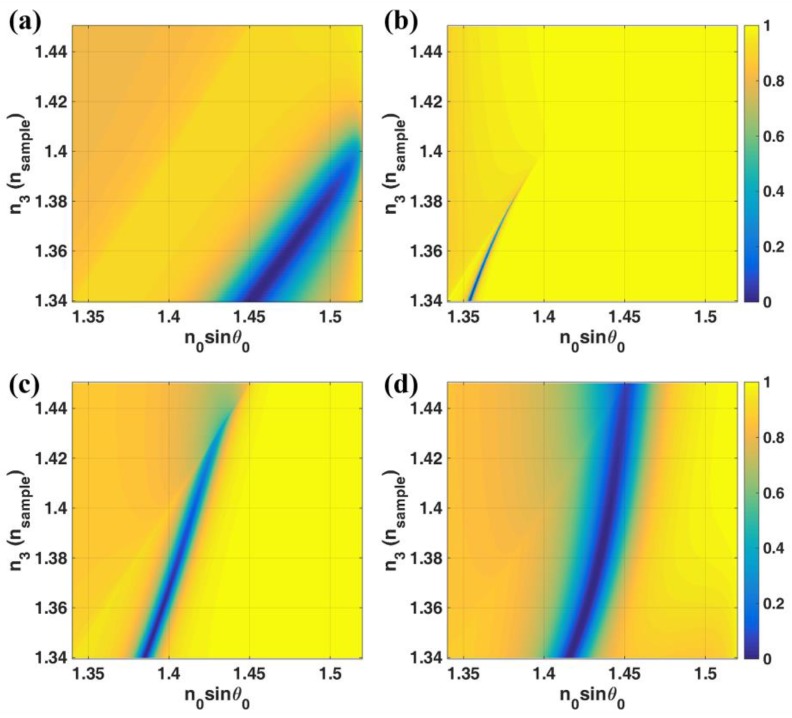
|*r_p_*|^2^ responses when the sample refractive index *n*_3_ changed from 1.34 to 1.45. (**a**) *n*_0_ = *n*_1_ = 1.52, *n*_2_ = 0.18344 + 3.4332*i* with *d*_2_ = 48 nm and varying *n*_3_; (**b**) *n*_0_ = 1.52, *n*_1_ = 1.34 with *d*_1_ = 930 nm, *n*_2_ = 0.18344 + 3.4332*i* with *d*_2_ = 20 nm and varying *n*_3_; (**c**) *n*_0_ = 1.52, *n*_1_ = 1.34 with *d*_1_ = 510 nm, *n*_2_ = 0.18344 + 3.4332*i* with *d*_2_ = 40 nm and varying *n*_3_; and (**d**) *n*_0_ = 1.52, *n*_1_ = 1.34 with *d*_1_ = 3600 nm, *n*_2_ = 0.18344 + 3.4332*i* with *d*_2_ = 60 nm and varying *n*_3_.

For LRSPP excited by evanescent wave coupling, the *n*_1_ of 1.34 was chosen as the cavity refractive index to ensure that all the sample refractive indices were below the cavity refractive index. The IIMI structures with different gold thicknesses *d*_2_ and fixed sample refractive index of 1.34 were then calculated by varying the cavity thickness *d*_1_ to determine the lowest reflectance intensity position for the LRSPP for each gold thickness. The operating points where the lowest intensity dips occurred for the gold thickness between 20 nm to 75 nm were shown in Figure 7. It can be seen from the Figure 2c that the cut-off of the plasmonic mode is around 20 nm of gold thickness for LRSPP excitation. The dip movement responses are shown in Figure 8b–d for the gold thicknesses of 20 nm, 40 nm and 60 nm, respectively. It can be seen from Figure 8a for the 20 nm thick that the plasmonic dip became very sharp with 21 times narrower FWHM boosting up to the *FoM* by 12 times, but it does lose half of the dip refractometric sensitivity compared to the Kretschmann case. The dynamic range for the 20 nm case was around 1.36595, which was very limited and also shorter than the Kretschmann case. This was due to the LRSPP coupling becoming inefficient when the refractive indices sandwiching the gold had a larger mismatch. An increase in the gold thickness can extend the dynamic range, but the dip refractometric sensitivity, the FWHM and consequently the *FoM* became worse. To cover the whole range of the sample refractive indices, the gold thickness of at least 60 nm was required and the *FoM* became 12.90102, which was almost two times worse than the Kretschmann setup.

**Figure 9:**
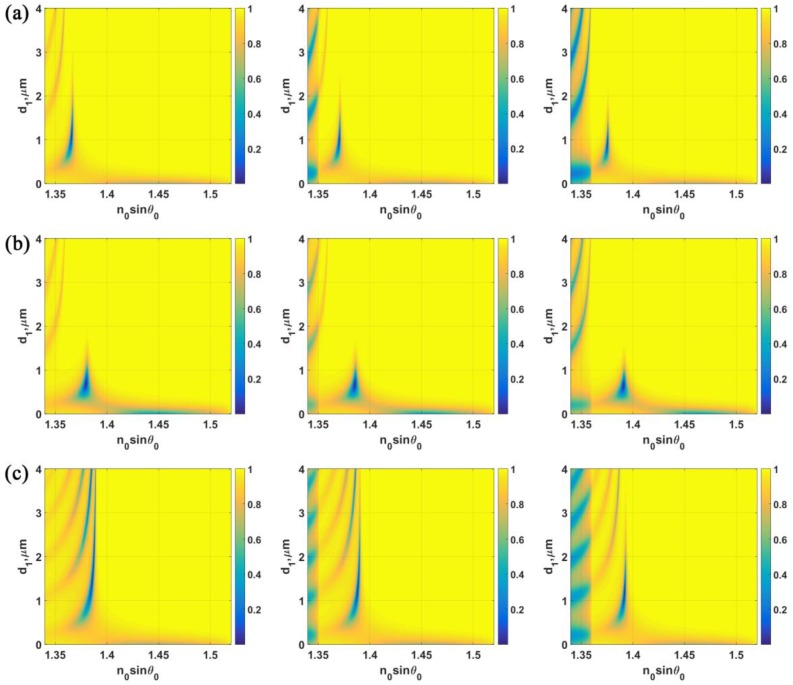

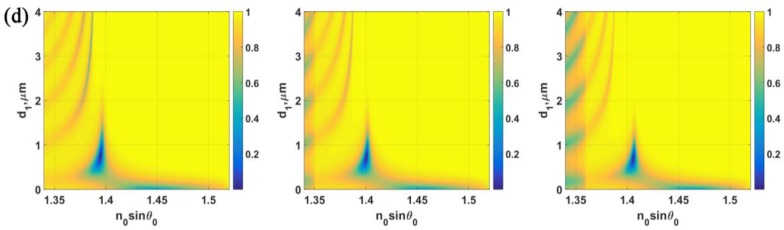
|*r_p_*|^2^ responses for the following structure parameters. (**a**) *n*_0_ = 1.52, *n*_1_ = 1.3616, *n*_2_ = 0.18344 + 3.4332*i* with *d*_2_ = 20 nm; (**b**) *n*_0_ = 1.52, *n*_1_ = 1.3616, *n*_2_ = 0.18344 + 3.4332*i* with *d*_2_ = 30 nm; (**c**) *n*_0_ = 1.52, *n*_1_ = 1.39, *n*_2_ = 0.18344 + 3.4332*i* with *d*_2_ = 20 nm; (**d**) *n*_0_ = 1.52, *n*_1_ = 1.39, *n*_2_ = 0.18344 + 3.4332*i* with *d*_2_ = 30 nm. Each column represents responses due to different sample refractive indices, *n*_3_ = 1.34, 1.35 and 1.36 shown in left, middle and right columns, respectively.

For the LRSPP excited through interferometric modes provided the resonant cavity, two refractive indices of the dielectric cavity were chosen in this study. The *n*_1_ refractive indices were 1.3616 (KF) and 1.3900 (LiF). The |*r_p_*|^2^ contours for different structure parameters are shown in Figure 9. To demonstrate different sensitivity responses at different cavity thicknesses *d*_1_ and refractive indices *n*_1_, the |*r_p_*|^2^ contours for *n*_3_ of 1.34, 1.35 and 1.36 shown in left, middle and right columns, respectively. Figure 9a,b shows the LRSPP structures with *n*_1_ of 1.3616 for *d*_2_ of 20 nm and 30 nm, respectively. Figure 9c,d shows the LRSPP structures with *n*_1_ of 1.3900 for *d*_2_ of 20 nm and 30 nm, respectively. The performance parameters for these four structures at 500 nm *d*_1_ thickness step were summarized in Table 1. It can be seen from the Table 1 that the LRSPP excited through the interferometric modes can be employed to extend the dynamic range of the LRSPP covering the whole sample refractive indices without losing too much of the dip refractometric sensitivity, the FWHM and the *FoM* such as (1) *n*_1_ of 1.3616 with *d*_1_ of 1000 nm and *d*_2_ of 20 nm and (2) *n*_1_ of 1.3900 with *d*_1_ of 1000 nm and *d*_2_ of 20 nm. It can be seen that these two example cases had a longer dynamic range than the 20 nm case of the LRSPP excited by the evanescent wave. 

The dynamic range can be extended further covering the whole range of the sample refractive indices by increasing the thickness of the gold to just 30 nm with *n*_1_ of 1.3900 and *d*_1_ of 500 nm whereas the gold thickness needed to be increased to 60 nm for the LRSPP excited by evanescent wave coupling case. The *FoM* for this 30 nm case is six times higher than the 60 nm gold for LRSPP excited by evanescent wave and three times higher than the Kretschmann case. 

The LRSPP excited by the interferometric mode can have a higher *FoM* than the LRSPP excited by evanescent wave coupling, however, it does have a very narrow dynamic range as shown in Table 1 for *n*_1_ of 1.3616 with *d*_1_ of 1500 nm and *d*_2_ of the 20 nm cases. It is interesting to note that for the same gold thickness as Kretschmann configuration of 48 nm the LRSPP excited by the resonant cavity *n*_1_ of 1.3900 and *d*_1_ of 500 nm has a longer dynamic range and three times narrower plasmonic dip but the dip refractometric sensitivity is less than the Kretschmann case. The plasmonic dips for the LRSPP cases appear at a lower *k*-vectors than the Kretschmann SPR.

### 3.5. Field Enhancement

**Figure 10:**
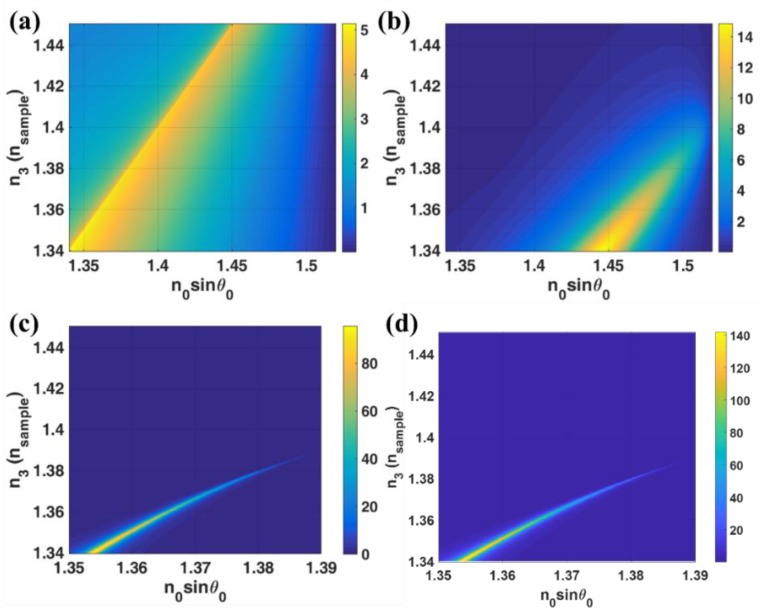
*|t_p_|*^2^ responses when the sample refractive index *n*_3_ changed from 1.34 to 1.45. (**a**) *n*_0_ = *n*_1_ = *n*_2_ = 1.52, and varying *n*_3_; (**b**) *n*_0_ = *n*_1_ = 1.52, *n*_2_ = 0.18344 + 3.4332*i* with *d*_2_ = 48 nm and varying *n*_3_; (**c**) *n*_0_ = 1.52, *n*_1_ = 1.34 with *d*_1_ = 930 nm, *n*_2_ = 0.18344 + 3.4332*i* with *d*_2_ = 20 nm and varying *n*_3_ and (**d**) *n*_0_ = 1.70, *n*_1_ = 1.34 with *d*_1_ = 930 nm, *n*_2_ = 0.18344 + 3.4332*i* with *d*_2_ = 20 nm and varying *n*_3_*.*

One key feature of surface plasmon resonance is the field enhancement on the surface of the metal. This unique feature is very useful for several applications including fluorescent excitation [42] and non-linear optics [43]. The field enhancement here was calculated as *|t_p_|*^2^ or the amount of transmitted light intensity on the metal layer. In this section, several layered structures were simulated and compared. The sample channel was again placed in the last dielectric layer with refractive indices ranging from 1.34 to 1.45, which cover a typical range for biological samples refractive indices [38,39]. Figure 10 shows *|t_p_|*^2^ for the different layered structure. A typical configuration for evanescent wave excitation in total internal reflection microscopy [44,45] was an interface between glass substrate and sample. The field enhancement *|t_p_|*^2^ for glass/sample interface calculated as shown in Figure 10a had the maximum value of 5.22. Figure 10b showed the conventional SPR excited using Kretschmann configuration. The maximum *|t_p_|*^2^ was 14.89, which was 2.8 times higher than the glass/sample interface. There were also a number of inconvenient issues for microscopy imaging. Firstly, *|t_p_|*^2^ values for each sample refractive index were not constant, forming a non-uniform field and secondly the SPR cannot cover the sample required refractive index range. Several recently developed microscopy techniques have included spatial light modulator devices such as an amplitude spatial light modulator [46], a phase spatial light modulator [47,48,49] or a digital micromirror device (DMD) [50]. Thirdly, they were excited at different *k*-vector positions making illumination control and difficulty in modulation. The same issues were also applied to the LRSPP excited by the evanescent wave as shown in Figure 10c,d. The 20 nm gold has been chosen since it can provide a decent amount of plasmons and a high-quality film can be fabricated. The maximum *|t_p_|*^2^ in Figure 10c was 6.45 times higher than the Kretschmann case shown in Figure 10a. The differences between the cases shown in Figure 10c,d was that the coupling index *n*_0_ where the Figure 10c case was 1.52 and the Figure 10d was 1.7. The *n*_0_ of 1.7 case had 1.48 times higher *|t_p_|*^2^ than the *n*_0_ of 1.52 case. This is because of the higher index contrast between *n*_0_ and *n*_1_ providing the higher evanescent wave enhancement. Note that the highest NA available in the market at the moment is the NA 1.7 APON100 × HOTIRF [51].

**Figure 11:**
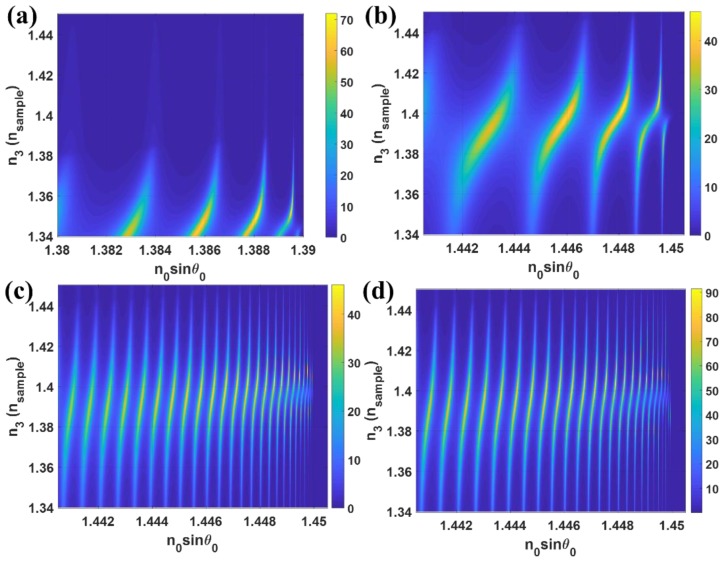
*t_p_|*^2^ responses when the sample refractive index *n*_3_ changed from 1.34 to 1.45. (**a**) *n*_0_ = 1.52, *n*_1_ = 1.39 with *d*_1_ = 1000 nm, *n*_2_ = 0.18344 + 3.4332*i* with *d*_2_ = 20 nm and varying *n*_3_; (**b**) *n*_0_ = 1.52, *n*_1_ = 1.45 with *d*_1_ = 1000 nm, *n*_2_ = 0.18344 + 3.4332*i* with *d*_2_ = 20 nm and varying *n*_3_; (**c**) *n*_0_ = 1.52, *n*_1_ = 1.45 with *d*_1_ = 5000 nm, *n*_2_ = 0.18344 + 3.4332*i* with *d*_2_ = 20 nm and varying *n*_3_ and (**d**) *n*_0_ = 1.7, *n*_1_ = 1.45 with *d*_1_ = 5000 nm, *n*_2_ = 0.18344 + 3.4332*i* with *d*_2_ = 20 nm and varying *n*_3_*.*

Having explained in the earlier section, the properties of the LRSPP excited by the interferometer modes can be engineered through the resonant cavity parameters *d*_1_ and *n*_1_. The sample refractive index dynamic range of the field enhancement can be controlled by the cavity refractive index *n*_1_ as shown in Figure 11a,b. Both of the structures in Figure 11a,b had the same cavity thickness *d*_1_ of 1000 nm, the difference between the two structures was that the *n*_1_ refractive indices for Figure 11a,b was 1.39 and 1.45 (silicon dioxide: SiO_2_ [52]), respectively. The field enhancement in n_1_ of 1.39 case was much greater than the *n*_1_ of 1.45 case, however, the dynamic range was much shorter. Having explained in Table 1 that the structure with the thicker cavity thickness had lower sensitivity to the refractive index change, this was also applicable to the structure design for field enhancement application. Figure 11c showed the *|t_p_|*^2^ responses for the cavity with *n*_1_ of 1.45 and *d*_1_ of 5000 nm. It can be seen that the *k*-vector position for each interferometric orders that excited the LRSPP became more linear than the thinner cavity case and independent of the sample refractive index. The magnitude of the field enhancement of 46.04 in the LRSPP excited through the interferometric mode was independent of the cavity thickness since the LRSPP was excited by the propagating wave in the cavity. Figure 11d showed the effect of the coupling refractive index *n*_0_ of 1.7. The higher coupling refractive case can enhance the field enhancement to 91.41 which was at least 5 times more than the conventional Kretschmann case.

### 3.6. Self-Referenced SPR System

**Figure 12:**
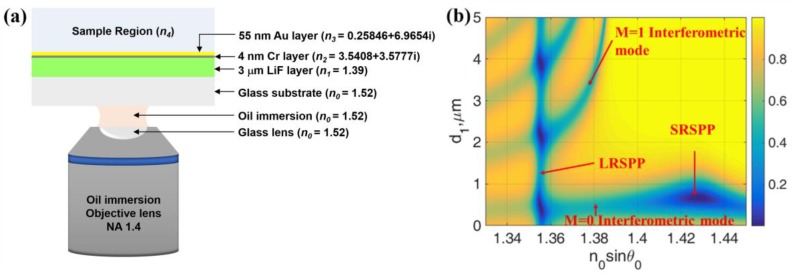
(**a**) The proposed self-reference SPR sensor (**b** structure and) |*r_p_*|^2^ responses when the sample refractive index n_4_ was 1.33 calculated for 1.064 μm incident wavelength.

We have demonstrated in the previous section that the interferometric modes can be designed so that it does not excite the LRSPP, therefore they will not be sensitive to the sample region. On the other hand, they can also excite the LRSPP and these LRSPPs can be more sensitive than the LRSPP excited by the evanescent wave coupling as shown in Table 1. This feature enables us to design a self-referenced SPR sensor having two reflectance dips, which the dip position movement due to the variations in the sensor can be distinguished from the signal change due to the sample refractive index change. The variations in the sensor are, for example, temperature fluctuation in the sensor, evaporation of oil immersion liquid. Figure 12a showed our proposed structure, which the structure was readily integratable on an oil immersion microscope. The incident wavelength of 1.064 μm was used in this section allowing the LRSPP to be excited at a lower incident *k*-vector to ensure that 1.4 NA can accommodate all the two modes. The proposed structure consisted of the following layers: glass substrate (*n*_0_ = 1.52), 3 μm thick of LiF with 1.39 refractive index (*n*_1_), 4 nm thick of Cr layer with 3.5408 + 3.5777*i* [25] refractive index (*n*_2_) (this also served as adhesion layer between gold and glass), 55 nm of gold layer with refractive index of 0.25846 + 6.9654*i* [25] (*n*_3_) and the sample channel with refractive index *n*_4_ was placed on the top of the sensor. Figure 12b showed |*r_p_*|^2^ response when the cavity thickness *d*_1_ was varied from 0 μm to 5 μm. It can be seen from the figure that at *d*_1_ of 3 μm the LRSPP occurred at the incident *k*-vector lower than the 1st order interferometric mode. The two modes were well separated enough so that the interferometric mode was independent of the LRSPP as explained in the earlier section.

**Figure 13:**
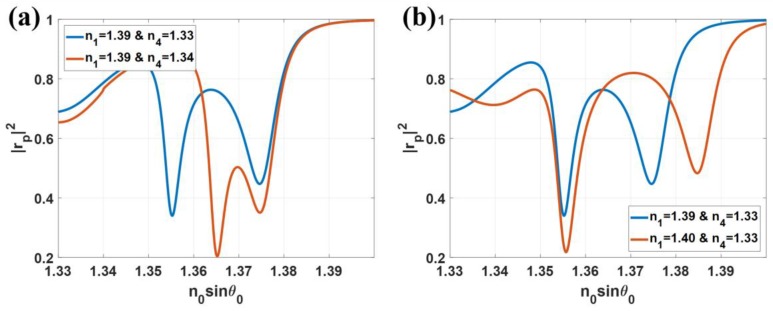
(**a**) |*r_p_*|^2^ responses when *n*_1_ = 1.39, *n*_4_ = 1.33 (blue curve) and 1.34 (red curve) and (**b**) |*r_p_*|^2^ responses when *n*_1_ = 1.39 (blue curve) and 1.40 (red curve) and *n*_4_ = 1.33.

Figure 13a shows the |*r_p_*|^2^ responses for when the refractive index of the sample changed from 1.33 to 1.34 depicted in blue curve for 1.33 and red curve for 1.34. Note that only the LRSPP dip moved to the higher *k*-vector when the sample refractive index changed. The 1st order interferometric dip did not move. Figure 13b shows the case where the refractive index in the illumination part changed and the sample refractive index was fixed, we mimicked this scenario by varying the index of the *n*_1_ from 1.39 to 1.4. It can be observed from the figure that the interferometric dip moved whereas the LRSPP did not when the refractive index of the sensor structure changed. This allows us to separate the change due to the illumination structure and the change due to the sample. This unique feature is not available in other structures such as IMIM structure.

## 4. Conclusions

In this paper, we have developed a theoretical framework to understand unique characteristics of IIMI structure under the following conditions: (1) the refractive index of the second dielectric layer was large than refractive index of the last dielectric layer and (2) when the light propagated in the second dielectric layer and was evanescent in the last dielectric layer. These conditions allowed multiple reflections in the second dielectric layer and an embedded interferometric detection. The IIMI structure under the conditions can be employed to enhance the LRSPP excitation, sensitivity, field enhancement and extend the dynamic range for LRSPP excitation. We have proposed structures for: (1) *FoM* enhancement for refractive index sensing application and (2) self-reference structure allowing the signal change due to sensor structure and the signal change due to the sample refractive index change to be distinguished. This feature is only available in the proposed IIMI structure.

## Figures and Tables

**Table 1:** The performance evaluation parameters for SPR excited by Kretschmann configuration, LRSPP excited through evanescent wave coupling and LRSPP excited by interferometric mode.

Structure Parameters *n*_1_, *d*_1_, *d*_2_ (in nm)	Dynamic Range (D)	*n_0_sinθ_0_* at Lower D Limit	*n_0_sinθ*_0_ at Upper D Limit	Sensitivity (S)	FWHM at |*r_p_*|^2^ = 0	*n_0_sinθ*_0_ Where the FWHM Calculated	*FoM*	Shown in Figure
**Kretschmann Configuration**								
1.5200, 0, 48	1.3400–1.3983	1.4510	1.5137	1.0757	0.0469	1.3400	22.9344	8a
**LRSPP excited by evanescent wave coupling**								
1.3400, 930, 20	1.3400–1.3660	1.3538	1.3697	0.6127	0.0022	1.3400	273.6243	8b
1.3400, 660, 30	1.3400–1.3885	1.3685	1.3960	0.5680	0.0056	1.3400	101.8497	-
1.3400, 510, 40	1.3400–1.4191	1.3849	1.4247	0.5030	0.0108	1.3400	46.4032	8c
1.3400, 420, 50	1.3400–1.4485	1.4010	1.4485	0.4380	0.0176	1.3400	24.9548	-
1.3400, 360, 60	1.3400–1.4500	1.4155	1.4516	0.3276	0.0254	1.3400	12.9010	8d
1.3400, 310, 70	1.3400–1.4500	1.4284	1.4573	0.2621	0.0371	1.3400	7.0632	-
**LRSPP excited by interferometric mode**								
1.3616, 500, 20	1.3955–1.4157	1.3971	1.4151	0.8927	0.0165	1.4092	54.1772	9a
1.3616, 1000, 20	1.3400–1.3778	1.3655	1.3858	0.5374	0.0024	1.3709	223.4523	9a
1.3616, 1500, 20	1.3400–1.3404	1.3665	1.3667	0.4198	0.0011	1.3665	373.7145	9a
1.3616, 500, 30	1.3400–1.4439	1.3783	1.4439	0.6315	0.0072	1.4207	87.5131	9b
1.3900, 500, 20	1.4218–1.4477	1.4249	1.4474	0.8673	0.0122	1.4412	71.0818	9c
1.3900, 1000, 20	1.3400–1.4029	1.3829	1.4133	0.4836	0.0026	1.3984	182.3115	9c
1.3900, 1500, 20	1.3400–1.3652	1.3860	1.3947	0.3456	0.0026	1.3872	132.2752	9c
1.3900, 2000, 20	1.3400–1.3512	1.3873	1.3901	0.2490	0.0018	1.3873	138.0813	9c
1.3900, 2500, 20	1.3400–1.3454	1.3881	1.3891	0.1840	0.0012	1.3881	154.6768	9c
1.3900, 3000, 20	1.3400–1.3425	1.3885	1.3889	0.1391	0.0008	1.3885	170.0563	9c
1.3900, 3500, 20	1.3400–1.3408	1.3888	1.3889	0.1073	0.0006	1.3888	185.0506	9c
1.3900, 4000, 20	1.3400–1.3397	1.3890	1.3890	0.0840	0.0004	1.3890	199.8070	9c
1.3900, 500, 30	1.3400–1.4500	1.3921	1.4570	0.5903	0.0077	1.4479	76.5967	9d
1.3900, 1000, 30	1.3400–1.3477	1.3960	1.4001	0.5388	0.0042	1.3960	127.6527	9d
1.3900, 500, 40	1.3400–1.4500	1.4093	1.4726	0.5754	0.0137	1.4170	42.0868	-
1.3900, 500, 48	1.3400–1.4067	1.4214	1.4655	0.6605	0.0138	1.4214	48.0453	-

## Supplementary Materials

The following are available online at http://www.mdpi.com/1424-8220/18/9/2757/s1, Figure S1: |*r_p_*|^2^ responses using silver as the metal layer, Table S1: The performance evaluation for the silver material, Figure S2: |*r_p_*|^2^ responses for the incident wavelength of 785 nm, Table S2: The performance evaluation for the incident wavelength of 785 nm, Figure S3: |*r_p_*|^2^ responses for the incident wavelength of 1024 nm, Table S3: The performance evaluation for the incident wavelength of 1024 nm.
