# Supplementary material for: Enhancement of Long-Range Surface Plasmon Excitation, Dynamic Range and Figure of Merit Using a Dielectric Resonant Cavity
Source: Sensors (Basel). 2018 Aug 22;18(9):2757. doi: 10.3390/s18092757 (PMC6165055; doi:10.3390/s18092757)
Supplement: Supplementary file 1 [file sensors-18-02757-s001.pdf]

Supplementary Information

Enhancement of Long-Range Surface Plasmons Excitation, Dynamic Range and Figure of Merit Using Dielectric Resonant Cavity

Phitsini Suvarnaphaet † and Suejit Pechprasarn *,†

College of Biomedical Engineering, Rangsit University, Pathum Thani 12000, Thailand; phitsini.s@rsu.ac.th

* Correspondence: suejit.p@rsu.ac.th; Tel.: +66-2-997-2200 (ext. 1469)

† These authors contributed equally to this work.

In the study, gold has been chosen as the plasmonic layer of the SPR sensor for the reasons described in the main manuscript. Here, we have also quantified the performance of silver with refractive index 0.056206 + 4.2776*i* [25] as follows.

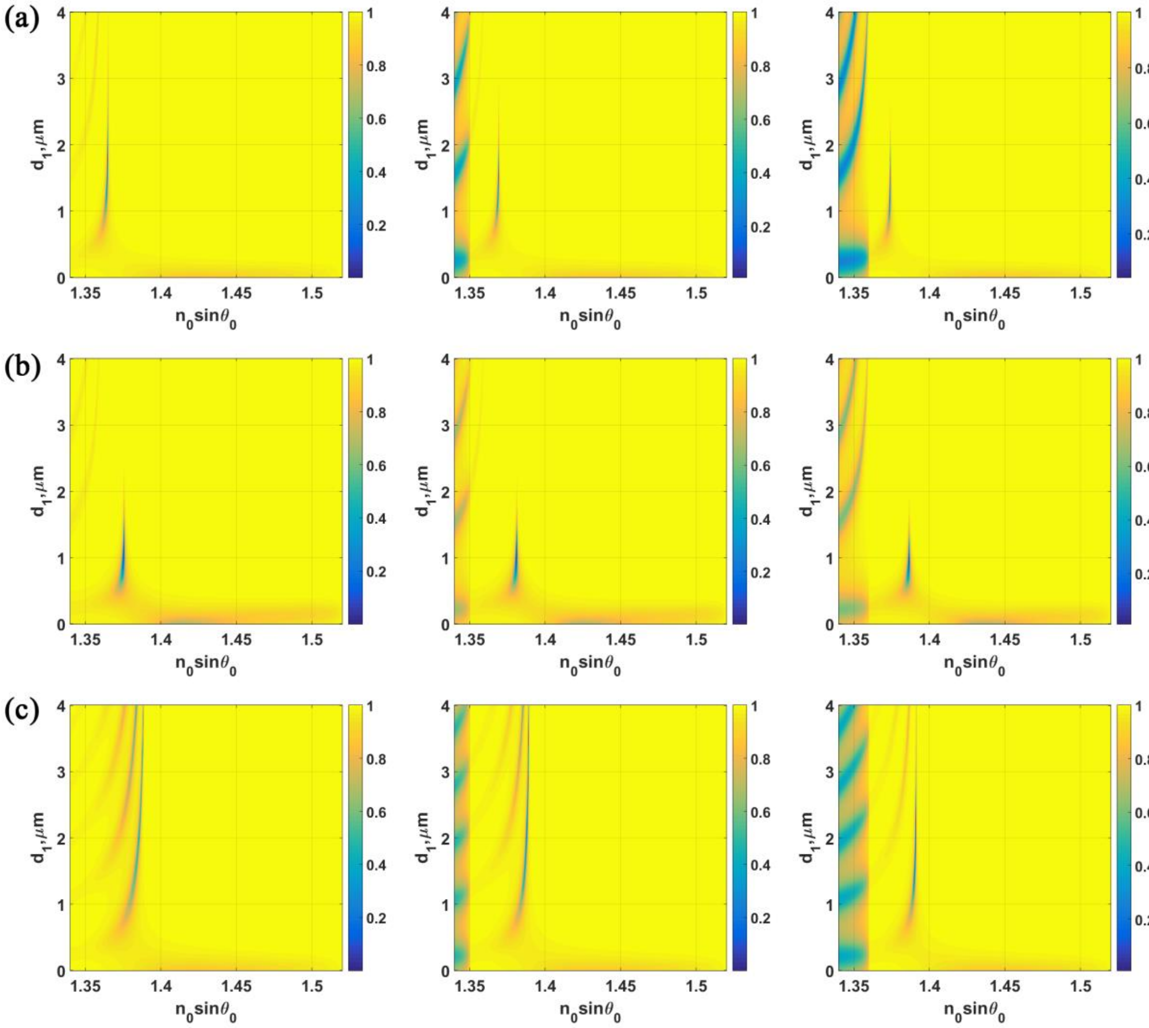

Figure S1. *Cont.*

(d)

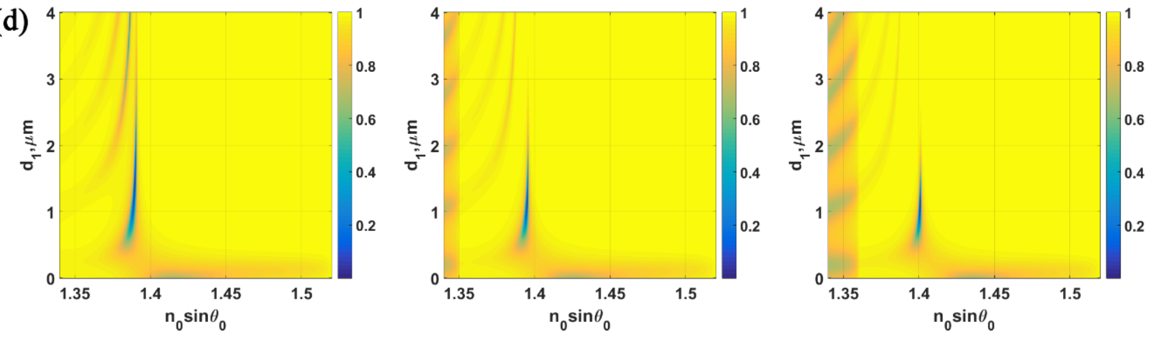

Figure S1. $|r_p|^2$ responses for the following structure parameters using silver as the metal layer. (**a**) $n_0 = 1.52$, $n_1 = 1.3616$, $n_2 = 0.056206 + 4.2776i$ with $d_2 = 20$ nm. (**b**) $n_0 = 1.52$, $n_1 = 1.3616$, $n_2 = 0.056206 + 4.2776i$ with $d_2 = 30$ nm. (**c**) $n_0 = 1.52$, $n_1 = 1.39$, $n_2 = 0.056206 + 4.2776i$ with $d_2 = 20$ nm. (**d**) $n_0 = 1.52$, $n_1 = 1.39$, $n_2 = 0.056206 + 4.2776i$ with $d_2 = 30$ nm. Each column represents responses due to different sample refractive index, n_3 = 1.34, 1.35 and 1.36 shown in left, middle and right columns, respectively. Note that λ = 633 nm.

Table S1. The performance evaluation parameters for SPR excited by Kretschmann configuration, LRSPP excited through evanescent wave coupling and LRSPP excited by interferometric mode using silver.

Structure Parameters n_1, d_1, d_2 (in nm)	Dynamic Range (D)	$n_0 sin\theta_0$ at Lower D Limit	$n_0 sin\theta_0$ at Upper D Limit	Sensitivity (S)	FWHM at $\|r_p\|^2 = 0$	$n_0 sin\theta_0$ Where the FWHM Calculated	*FoM*
**Kretschmann Configuration**							
1.5200, 0, 55	1.3400–1.4355	1.4089	1.5192	1.1550	0.0083	1.4111	139.1535
**LRSPP excited by evanescent wave coupling**							
1.3400, 930, 30	1.3400–1.3800	1.3638	1.3851	0.5325	0.0012	1.3643	443.7500
1.3400, 660, 50	1.3400–1.4105	1.3851	1.4083	0.3291	0.0034	1.3861	96.7877
1.3400, 510, 78	1.3400–1.4500	1.4010	1.4140	0.1182	0.0072	1.4030	16.4141
1.3400, 420, 100	1.3400–1.3470	1.4070	1.4200	1.8571	0.0162	1.4113	114.6384
1.3400, 360, 92	1.3400–1.3540	1.4080	1.4280	1.4286	0.0179	1.4111	79.8085
1.3400, 310, 86	1.3400–1.3592	1.4084	1.4397	1.6302	0.0171	1.4112	95.3338
**LRSPP excited by interferometric mode**							
1.3616, 500, 61	1.3400–1.4500	1.4015	1.4039	0.0218	0.0056	1.4032	3.8961
1.3616, 1000, 32	1.3400–1.3796	1.3773	1.3992	0.5530	0.0014	1.3776	395.0216
1.3616, 1500, 20	1.3400–1.3681	1.3643	1.3683	0.1423	0.0006	1.3645	237.2479
1.3616, 500, 60	1.3400–1.4500	1.4010	1.4390	0.3455	0.0055	1.4026	62.8099
1.3900, 500, 50	1.3400–1.4500	1.4012	1.4649	0.5791	0.0060	1.4028	96.5152
1.3900, 1000, 35	1.3400–1.3864	1.3922	1.4224	0.6509	0.0026	1.3929	250.3316
1.3900, 1500, 29	1.3400–1.3563	1.3886	1.3978	0.5644	0.0016	1.3890	352.7607
1.3900, 2000, 26	1.3400–1.3511	1.3877	1.3924	0.4234	0.0011	1.3879	384.9304
1.3900, 2500, 25	1.3400–1.3477	1.3879	1.3904	0.3247	0.0008	1.3881	405.8442
1.3900, 3000, 25	1.3400–1.3448	1.3884	1.3897	0.2708	0.0006	1.3884	451.3889
1.3900, 3500, 25	1.3400–1.3431	1.3888	1.3893	0.1613	0.0005	1.3888	322.5806
1.3900, 4000, 25	1.3400–1.3423	1.3890	1.3893	0.1304	0.0003	1.3890	434.7826
1.3900, 500, 54	1.3400–1.4500	1.4033	1.4671	0.5800	0.0057	1.4048	101.7544
1.3900, 1000, 30	1.3400–1.4093	1.3879	1.4277	0.5743	0.0026	1.3884	220.8902
1.3900, 500, 45	1.4152–1.4500	1.4491	1.4617	0.3621	0.0059	1.3994	61.3676
1.3900, 500, 48	1.3400–1.4500	1.4001	1.4638	0.5791	0.0060	1.4015	96.5152

Other wavelengths for SPR excitation in the near-infrared region, i.e., 785 nm and 1024 nm are also presented in Figure S2 and Figure S3, respectively, and the sensing performance is summarized in Table S2 and Table S3.

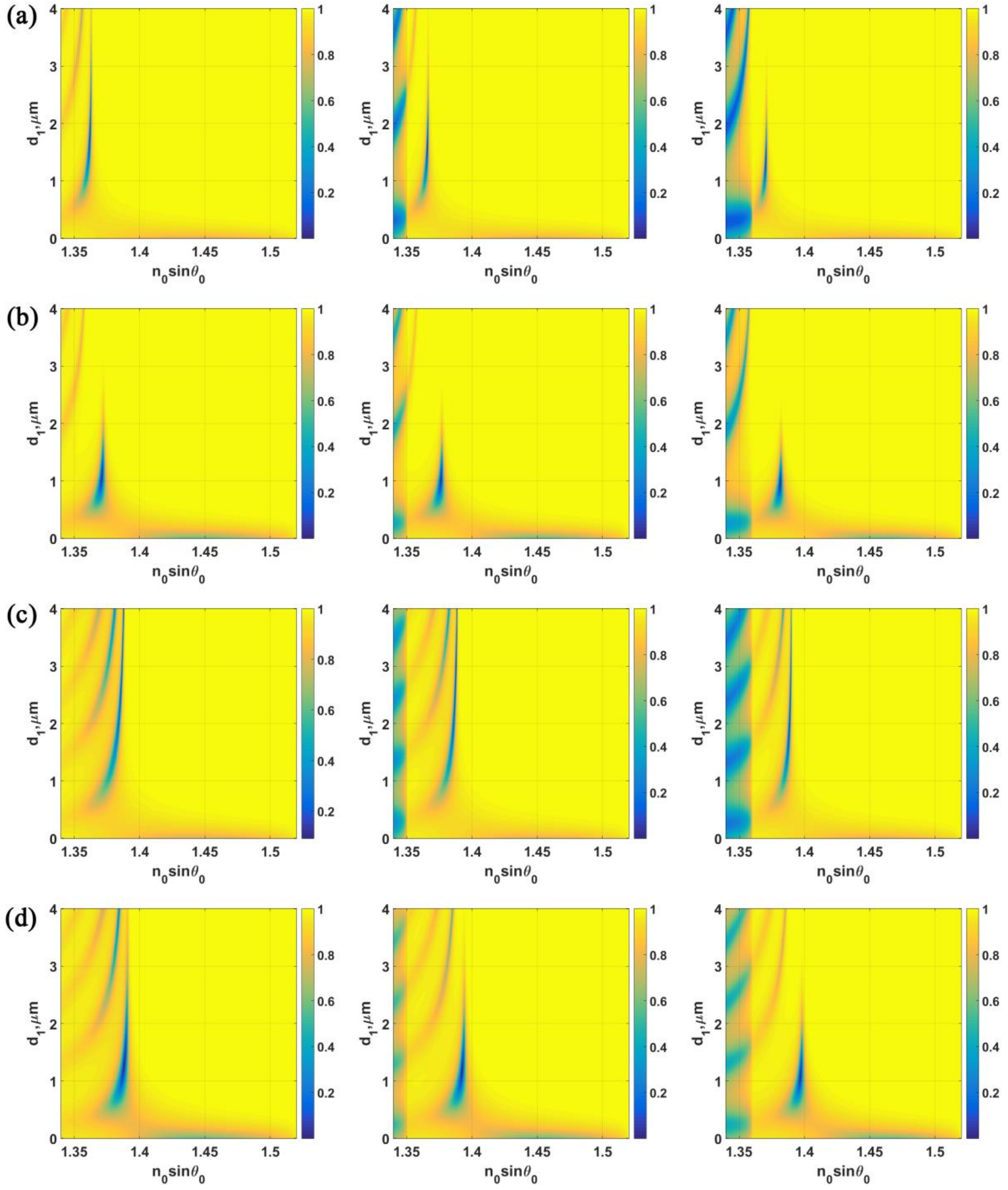

Figure S2. $|r_p|^2$ responses for the following structure parameters using λ = 785 nm. (**a**) n_0 = 1.52, n_1 = 1.3616, n_2 = 0.18344 + 3.4332i with d_2 = 20 nm. (**b**) n_0 = 1.52, n_1 = 1.3616, n_2 = 0.18344 + 3.4332*i* with d_2 = 30 nm. (**c**) n_0 = 1.52, n_1 = 1.39, n_2 = 0.18344 + 3.4332*i* with d_2 = 20 nm. (**d**) n_0 = 1.52, n_1 = 1.39, n_2 = 0.18344 + 3.4332*i* with d_2 = 30 nm. Each column represents responses due to different sample refractive index, n_3 = 1.34, 1.35 and 1.36 shown in left, middle and right columns, respectively.

Table S2. The performance evaluation parameters for SPR excited by Kretschmann configuration, LRSPP excited through evanescent wave coupling and LRSPP excited by interferometric mode using λ = 785 nm.

Structure Parameters n_1, d_1, d_2 (in nm)	Dynamic Range (D)	$n_0 sin\theta_0$ at Lower D Limit	$n_0 sin\theta_0$ at Upper D Limit	Sensitivity (S)	FWHM at $\|r_p\|^2 = 0$	$n_0 sin\theta_0$ Where the FWHM Calculated	*FoM*
**Kretschmann Configuration**							
1.5200, 0, 48	1.3400–1.4134	1.4361	1.5171	1.1035	0.0543	1.4480	20.3231
**LRSPP excited by evanescent wave coupling**							
1.3400, 930, 32	1.3400–1.3772	1.3609	1.3837	0.6129	0.0040	1.3620	153.2258
1.3400, 660, 47	1.3400–1.4134	1.3787	1.4196	0.5572	0.0097	1.3814	57.4454
1.3400, 510, 40	1.3400–1.4496	1.3958	1.4496	0.4909	0.0183	1.4010	26.8238
1.3400, 420, 80	1.3400–1.4500	1.4131	1.4627	0.4509	0.0294	1.4214	15.3370
1.3400, 360, 90	1.3400–1.4500	1.4212	1.4730	0.4709	0.0509	1.4322	9.2517
1.3400, 310, 130	1.3400–1.4500	1.4420	1.4869	0.4082	0.0741	1.4715	5.5085
**LRSPP excited by interferometric mode**							
1.3616, 500, 62	1.3400–1.4500	1.4070	1.4684	0.5582	0.0192	1.4124	29.0720
1.3616, 1000, 33	1.3400–1.3821	1.3735	1.3979	0.5796	0.0046	1.3747	125.9940
1.3616, 1500, 23	1.3400–1.3641	1.3638	1.3760	0.5062	0.0022	1.3643	230.1018
1.3616, 500, 60	1.3400–1.4500	1.3926	1.4584	0.5982	0.0166	1.3964	36.0350
1.3900, 500, 50	1.3400–1.4500	1.4162	1.4961	0.7264	0.0208	1.4220	34.9213
1.3900,1000,35	1.3400–1.3959	1.3904	1.4230	0.5832	0.0070	1.3924	83.3120
1.3900, 1500, 28	1.3400–1.3691	1.3861	1.3998	0.4708	0.0039	1.3872	120.7155
1.3900, 2000, 25	1.3400–1.3599	1.3857	1.3929	0.3618	0.0025	1.3864	144.7236
1.3900, 2500, 24	1.3400–1.3535	1.3866	1.3902	0.2667	0.0018	1.3870	148.1481
1.3900, 3000, 23	1.3400–1.3518	1.3872	1.3895	0.1949	0.0012	1.3873	162.4294
1.3900, 3500, 22	1.3400–1.3523	1.3875	1.3893	0.1463	0.0009	1.3877	162.6016
1.3900, 4000, 23	1.3400–1.3468	1.3881	1.3890	0.1324	0.0007	1.3882	189.0756
1.3900, 500, 70	1.3400–1.4369	1.4282	1.4930	0.6687	0.0191	1.4323	35.0121
1.3900, 1000, 30	1.3400–1.4151	1.3849	1.4269	0.5593	0.0067	1.3864	83.4708
1.3900, 500, 50	1.3400–1.4500	1.4044	1.4781	0.6700	0.0195	1.4094	34.3590
1.3900, 500, 48	1.3400–1.4500	1.4023	1.4753	0.6636	0.0188	1.4066	35.2998

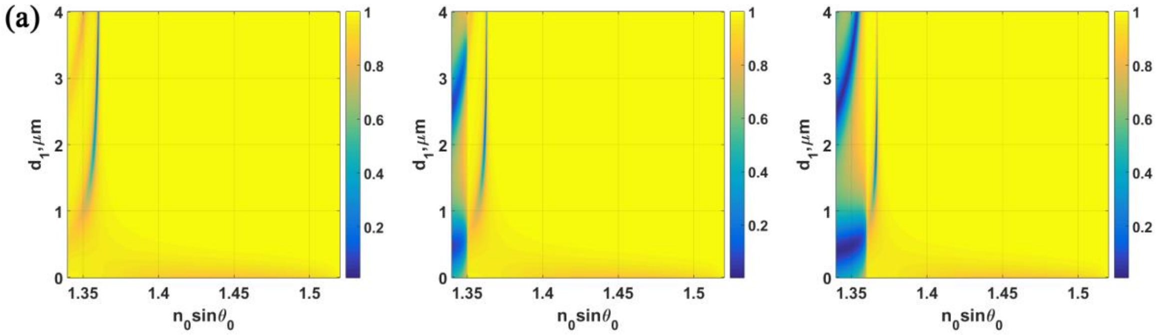

Figure S3. *Cont.*

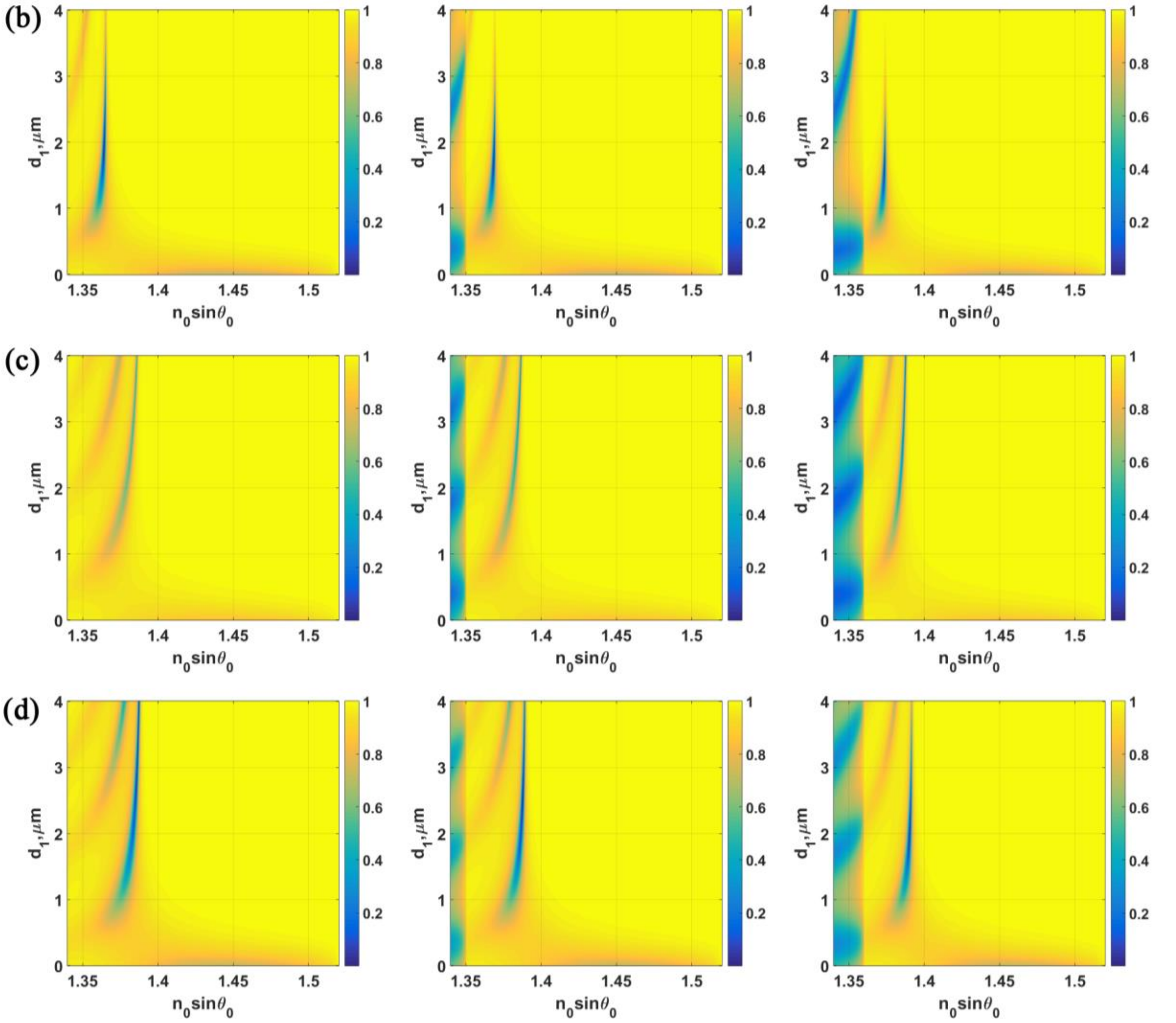

Figure S3. $|r_p|^2$ responses for the following structure parameters using λ = 1024 nm. (**a**) n_0 = 1.52, n_1 = 1.3616, n_2 = 0.18344 + 3.4332*i* with d_2 = 20 nm. (**b**) n_0 = 1.52, n_1 = 1.3616, n_2 = 0.18344 + 3.4332*i* with d_2 = 30 nm. (**c**) n_0 = 1.52, n_1 = 1.39, n_2 = 0.18344 + 3.4332i with d_2 = 20 nm. (**d**) n_0 = 1.52, n_1 = 1.39, n_2 = 0.18344 + 3.4332*i* with d_2 = 30 nm. Each column represents responses due to different sample refractive index, n_3 = 1.34, 1.35 and 1.36 shown in left, middle and right columns, respectively.

Table S3. The performance evaluation parameters for SPR excited by Kretschmann configuration, LRSPP excited through evanescent wave coupling and LRSPP excited by interferometric mode using λ = 1024 nm.

Structure Parameters n_1, d_1, d_2 (in nm)	Dynamic Range (D)	$n_0 \sin\theta_0$ at Lower D Limit	$n_0 \sin\theta_0$ at Upper D Limit	Sensitivity (S)	FWHM at $\|r_p\|^2 = 0$	$n_0 \sin\theta_0$ Where the FWHM Calculated	*FoM*
**Kretschmann Configuration**							
1.5200, 0, 78	1.3979	1.4394	1.5141	1.2902	0.0470	1.4511	27.4501
**LRSPP excited by evanescent wave coupling**							
1.3400, 930, 60	1.3974	1.3778	1.4104	0.5679	0.0082	1.3801	69.2615
1.3400, 660, 80	1.4498	1.3949	1.4498	0.5000	0.0184	1.4001	27.1739
1.3400, 510, 110	1.4500	1.4167	1.4673	0.4600	0.0348	1.4261	13.2184
1.3400, 420, 100	1.4500	1.4135	1.4736	0.5464	0.0425	1.4232	12.8556

Table S3. *Cont.*

Structure Parameters n_1, d_1, d_2 (in nm)	Dynamic Range (D)	$n_0 sin\theta_0$ at Lower D Limit	$n_0 sin\theta_0$ at Upper D Limit	Sensitivity (S)	FWHM at $\lvert r_p \rvert^2 = 0$	$n_0 sin\theta_0$ Where the FWHM Calculated	*FoM*
**LRSPP excited by interferometric mode**							
1.3400, 360, 90	1.4500	1.4530	1.4770	0.4715	0.0478	1.4246	9.8643
1.3616, 500, 95	1.3400–1.4500	1.4181	1.4856	0.6136	0.0311	1.4264	19.7311
1.3616, 1000, 55	1.3400–1.4048	1.3841	1.4224	0.5910	0.0079	1.3863	74.8164
1.3616, 1500, 23	1.3400–1.3809	1.3667	1.3898	0.5648	0.0034	1.3675	166.1153
1.3616, 500, 100	1.3400–1.4500	1.4217	1.4879	0.6018	0.0317	1.4302	18.9848
1.3900, 500, 90	1.3400–1.4500	1.4248	1.5076	0.7527	0.0304	1.4327	24.7608
1.3900, 1000, 60	1.3400–1.4059	1.4025	1.4449	0.6434	0.0104	1.4052	61.8653
1.3900, 1500, 40	1.3400–1.3910	1.3866	1.4143	0.5431	0.0057	1.3882	95.2872
1.3900, 2000, 35	1.3400–1.3721	1.3853	1.3998	0.4517	0.0038	1.3862	118.8719
1.3900, 2500, 38	1.3400–1.3461	1.3888	1.3915	0.4426	0.0024	1.3891	184.4262
1.3900, 3000, 30	1.3400–1.3605	1.3857	1.3915	0.2829	0.0021	1.3860	134.7271
1.3900, 3500, 30	1.3400–1.3555	1.3865	1.3900	0.2258	0.0016	1.3870	141.1290
1.3900, 4000, 30	1.3400–1.3512	1.3871	1.3893	0.1964	0.0012	1.3875	163.6905
1.3900, 500, 89	1.3400–1.4500	1.4241	1.5070	0.7536	0.0304	1.4328	24.7907
1.3900, 1000, 55	1.3400–1.4231	1.3973	1.4484	0.6149	0.0105	1.4003	58.5640
1.3900, 500, 80	1.3400–1.4500	1.4172	1.4995	0.7482	0.0297	1.4248	25.1913
1.3900, 500, 100	1.3400–1.4500	1.4323	1.5124	0.7282	0.0289	1.4392	25.1966
